# Endoreversible Stirling Cycles: Plasma Engines at Maximal Power

**DOI:** 10.3390/e27080807

**Published:** 2025-07-28

**Authors:** Gregory Behrendt, Sebastian Deffner

**Affiliations:** 1 Department of Physics, University of Maryland, Baltimore County, Baltimore, MD 21250, USA; deffner@umbc.edu; 2 Quantum Science Institute, University of Maryland, Baltimore County, Baltimore, MD 21250, USA; 3 National Quantum Laboratory, College Park, MD 20740, USA

**Keywords:** plasma engine, endoreversible Stirling cycle

## Abstract

Endoreversible engine cycles are a cornerstone of finite-time thermodynamics. We show that endoreversible Stirling engines operating with a one-component plasma as a working medium run at maximal power output with the Curzon–Ahlborn efficiency. As a main result, we elucidate that this is actually a consequence of the fact that the caloric equation of state depends only linearly on temperature and only additively on volume. In particular, neither the exact form of the mechanical equation of state nor the full fundamental relation are required. Thus, our findings immediately generalize to a larger class of working plasmas, far beyond simple ideal gases. In addition, we show that for plasmas described by the photonic equation of state, the efficiency is significantly lower. This is in stark contrast to endoreversible Otto cycles, for which photonic engines have an efficiency larger than the Curzon–Ahlborn efficiency.

## 1. Introduction

Among all the various states of matter, plasmas have a unique place. In particular, quantum plasmas exist only in the most extreme environments, such as the interior of stars, in the early universe, or more mundanely in highly intense laser fields [1]. In its simplest form, a plasma is a superheated gas, in which electrons have been stripped from atoms, creating a mixture of positively charged ions and negatively charged electrons [1]. It provides the circumstances for nuclear fusion, which is a nuclear reaction where two light atomic nuclei combine to form a heavier one, releasing a tremendous amount of energy [2]. The potential technological applications of nuclear fusion are tremendous, which is why its realization is considered one of the “14 Grand Challenges for Engineering in the 21st Century” [3].

From a thermodynamic point of view, nuclear reactions in plasmas can also be understood as heat engine cycles [4,5]. In fact, the electrostatic interactions within a plasma behave very akin to the usual mechanical pressure in typical gases [6]. Interestingly, the somewhat natural engine cycle for plasma engines is the Stirling cycle [7]. More broadly, technological applications of the Stirling cycle appear rather promising; see, for instance, the recent review [8]. The natural question thus arises what the efficiency of plasma Stirling engines is at maximal power output.

In the present work, we answer this question within the framework of endoreversible thermodynamics [9]. In endoreversible thermodynamics one assumes that all processes are slow enough that the system *locally equilibrates*, yet the processes are too fast for the system to reach a state of equilibrium with the environment. More specifically, imagine an engine, whose working medium is at equilibrium at temperature *T*. However, *T* is not equal to the temperature of the heat bath, $T_{\mathrm{bath}}$, and thus there is a temperature gradient at the boundaries of the engine. Now further imagine that the engine undergoes a slow, cyclic state transformation, where slow means that the working medium remains *locally* in equilibrium at all times. Then, from the point of view of the environment the device undergoes an irreversible cycle. Such state transformations are called *endoreversible* [9], which means that locally the transformation is reversible, but globally irreversible.

In a seminal work, Curzon and Ahlborn showed [10] that the efficiency of a Carnot engine undergoing an endoreversible cycle at maximal power is given by

(1) $$\eta_{\mathrm{CA}}=1-\sqrt{\frac{T_{c}}{T_{h}}}\;,$$

where $T_{c}$ and $T_{h}$ are the temperatures of the cold and hot reservoirs, respectively. Since its discovery the Curzon–Ahlborn efficiency (1) has received a great deal of attention. Also, see even earlier studies by Moutier [11] and Novikov [12], and related works by Rozonoer and Tsirlin [13,14,15]. More recently, it has been found, e.g., that endoreversible Otto [16] and Brayton [17] engines operating with ideal gases also have the same efficiency. However, it has also been shown that whether or not a finite-time Carnot cycle really assumes $\eta_{\mathrm{CA}}$ is determined by the “symmetry” of the dissipation [18] and on the specific form of the fundamental relation [19,20,21,22,23,24,25,26,27,28,29,30,31].

In the present work, we focus on plasma engines that run in endoreversible Stirling cycles. It is interesting to note that there are also several accounts in the literature of the fact that endoreversible Stirling cycles at maximum power operating with classical, ideal gases are described by $\eta_{\mathrm{CA}}$ [32,33,34]. Given that it is often a good assumption (in first order approximation) that plasmas can also be described as ideal gases [35], one is tempted to conclude that, clearly, plasma engines at maximal power also have the Curzon–Ahlborn efficiency. However, given that previous treatments of the endoreversible Stirling make, sometimes implicitly, often explicitly the assumption that the working medium is a regular, classical, ideal gas, it is not immediately obvious that plasmas do not require a separate treatment.

Therefore, we start with a detailed discussion on the necessary conditions under which the Curzon–Ahlborn efficiency arises. Particular emphasis is placed on a comprehensive and pedagogical derivation, which then also leads to immediate generalizations. In fact, we will see that any gases that are described by caloric equations of states that are linear in temperature and at most additive in volume lead to the Curzon–Ahlborn efficiency. Neither the mechanical equation of state nor the full fundamental relation is required.

This means, in particular, that endoreversible Stirling engines whose working plasmas require a second-order virial expansion also have the same efficiency. In the simplest case, their corresponding gas law is given by the van der Waals equation of state.

As an example of plasma engines that fall not within this class, we then analyze an engine that operates with an electron–positron–photon plasma. For high enough temperatures [36] such plasmas can be described by the photonic equation of state, which permits an almost completely analytical treatment. We find that photonic Stirling engines have a significantly smaller efficiency at maximal power. This is in stark contrast to Otto engines, in which case photonic working mediums lead to higher efficiency [25].

## 2. One-Component Plasmas—Modified Ideal Gas

We start with a Stirling engine that operates with a one-component plasma. For such situations one commonly assumes that the plasma can be treated as an “effective ideal gas” [35]. A classical, ideal gas is comprised of uniform, non-interacting, identical particles, whose caloric equation of state is proportional to temperature and independent of the volume:

(2) E=ςT.

The constant ς depends on natural constants and the number of degrees of freedom. For instance, for a classical ideal gas in three spatial dimensions we have [37], $\varsigma =3/2N\;k_{B}$, where *N* is the number of particles and $k_{B}$ is Boltzmann’s constant.

A one-component plasma is comprised of non-interacting, identical particles. At the fundamental level, a one-component plasma is nothing but a collection of uniform ions, that have been immersed in an equally and oppositely charged background, neutralizing the total charge of the plasma, and thus preventing any potential interparticle-Coulomb interactions [35]. Thus, it follows that the corresponding caloric equation of state for the one-component plasma is still directly proportional to the temperature, and all “quantum” modifications can be accounted for by an effective number of degrees of freedom along with an arrangement of phenomenological constants that are unique to that plasma. We can write

(3) $$E=E_{0}+f\;T$$

where $E_{0}$ is a constant off-setting the background energy, and *f* quantifies the effective degrees of freedom [35]. As we will see shortly, Equation (3) is a necessary and sufficient condition to obtain the Curzon–Ahlborn efficiency.

### 2.1. Endoreversible Cycle and Efficiency

In complete analogy to the endoreversible Carnot [10], Otto [16,24,25,26,27,28,29,30,31], and Brayton cycles [17], we now construct the endoreversible Stirling cycle.

**Figure 1:**
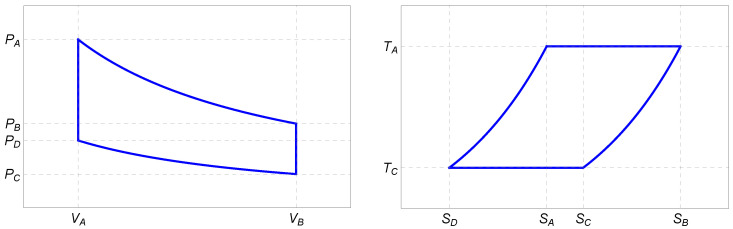
Schematic PV- and TS-diagrams of the Stirling cycle for the one-component plasma (3) as a working medium.

The Stirling cycle is a 4-stroke process comprising isothermal expansion, isochoric cooling, isothermal compression, and isochoric heating. The corresponding PV- and TS-diagrams for a one-component plasma (3) are depicted in Figure 1.

#### 2.1.1. A→B: Isothermal Expansion

During the hot isotherm, the plasma is in contact with a heat reservoir at temperature $T_{h}$. However, as usual in endoreversible thermodynamics [9], we assume that the plasma has not fully equilibrated with the hear reservoir, and rather has a temperature “slightly” below $T_{h}$, namely we have $T_{h,p}<T_{h}$. Fourier’s law in Newton’s form then dictates that the heat flux is linear in the temperature gradient [10], and we can write

(4) $$Q_{\mathrm{AB}}=\alpha \;t_{AB}\;\left(T_{h}-T_{h,p}\right)\;,$$

where α is the thermal conductivity and $t_{AB}$ is the duration of the stroke.

Now note that for one-component plasmas the internal energy is constant for isothermal processes; cf. Equation (3). Thus, we immediately have

(5) $$\Delta E=E_{B}-E_{A}\;\mathrm{and}\;W_{AB}=-Q_{AB},$$

namely the work produced during the isothermal expansion is equal and opposite in sign to the heat absorbed from the heat reservoir.

#### 2.1.2. B→C: Isochoric Cooling

During the isochoric stroke, the plasma is disconnected from the heat reservoirs. Thus, this stroke is identical to the ideal cycle. In any case, we have

(6) $$W_{BC}=0\;\mathrm{and}\;Q_{BC}=E_{B}-E_{C}=f\;\left(T_{B}-T_{C}\right)$$

where we used the caloric equation of state (3).

#### 2.1.3. C→D: Isothermal Compression

In complete analogy to the hot isotherm, during the cold, isothermal compression the plasma is in contact with a cold reservoir at temperature $T_{c}$. However, we again assume that the plasma has not fully equilibrated with the reservoir, and that its temperature, $T_{c,p}$, is slightly above $T_{c}$. Again, employing Fourier’s law, we can write

(7) $$Q_{CD}=\beta \;t_{CD}\;\left(T_{c}-T_{c,p}\right)$$

where β is the thermal conductivity for the cold stroke, and $t_{CD}$ is the duration. As before, we can also write

(8) $$\Delta E=E_{D}-E_{C}\;\mathrm{and}\;W_{CD}=-Q_{CD}\;,$$

which follows from the caloric equation of state (3).

#### 2.1.4. D→A: Isochoric Heating

The cycle is completed with another isochoric stroke. Again, the plasma is disconnected from the heat reservoirs. We have

(9) $$W_{DA}=0\;\mathrm{and}\;Q_{DA}=f\;\left(T_{D}-T_{A}\right)$$

#### 2.1.5. Endoreversible Efficiency

We are now interested in the efficiency at maximal power. To this end, consider that the plasma absorbs heat from the hot reservoir during the hot isotherm A→B. Thus, we can write

(10) $$\eta \equiv -\frac{W_{\mathrm{cyc}}}{Q_{AB}}=1+\frac{Q_{CD}}{Q_{AB}}\;,$$

where we used that the work produced during the entire cycle is $W_{\mathrm{cyc}}=-\left(Q_{AB}+Q_{CD}\right)$. Using Equations (4) and (7), we also have

(11) $$\eta =1+\frac{\beta \;t_{CD}}{\alpha \;t_{AB}}\;\frac{T_{c}-T_{c,p}}{T_{h}-T_{h,p}}\;,$$

which appears to suggest that the efficiency depends on the stroke times (see also Appendix A).

However, the expression for the efficiency (11) can be further simplified by employing the entropy balance over one cycle. In fact, we have

(12) $$0=\Delta S_{\mathrm{cyc}}=\Delta S_{AB}+\Delta S_{BC}+\Delta S_{CD}+\Delta S_{DA}\;.$$

Along the isothermal strokes, A→B and C→D, the entropy is simply given by the heat divided by temperature,

(13) $$\Delta S_{AB}=\frac{Q_{AB}}{T_{h,p}}=\frac{\alpha \;t_{AB}\;\left(T_{h}-T_{h,p}\right)}{T_{h,p}}\;\mathrm{and}\;\Delta S_{CD}=\frac{Q_{CD}}{T_{c,p}}=\frac{\beta \;t_{CD}\;\left(T_{c}-T_{c,p}\right)}{T_{c,p}}\;,$$

where we again used Equations (4) and (7).

For the isochoric strokes, we again exploit *only* the caloric equation of state (3). In its differential form, along the isochor, đW=0, we have dE=đQ=TdS. Thus, the total change in entropy can be written as

(14) $$\Delta S=\int_{S_{i}}^{S_{f}}\;dS=\int_{T_{i}}^{T_{f}}dT\;\frac{f}{T}\;=f\ln\left(\frac{T_{f}}{T_{i}}\right)\;.$$

Consequently, we obtain

(15) $$\Delta S_{BC}=-\Delta S_{DA}=f\;\ln\left(\frac{T_{c,p}}{T_{h,p}}\right)\;,$$

and thus,

(16) $$\frac{\alpha \;t_{AB}\;\left(T_{h}-T_{h,p}\right)}{T_{h,p}}=\frac{\beta \;t_{CD}\;\left(T_{c,p}-T_{c}\right)}{T_{c,p}}\;.$$

In other words, the efficiency (11) of an endoreversible Stirling cycle operating with a one-component plasma simply becomes

(17) $$\eta =1-\frac{T_{c,p}}{T_{h,p}}\;,$$

which is identical to the ideal efficiency [37] replacing the temperatures of the heat reservoirs with the corresponding temperatures of the plasma.

### 2.2. Efficiency at Maximal Power

As stated above, we are now interested in the efficiency at maximal power output. Inspecting Equation (17) we need to to determine the temperatures, $T_{h,p}$ and $T_{c,p}$, that maximize the power. As usual, we write

(18) $$P\equiv -\frac{W_{\mathrm{cyc}}}{\tau_{\mathrm{cyc}}}=\frac{Q_{AB}+Q_{CD}}{\gamma \;(t_{AB}+t_{CD})}\;,$$

where γ is a real constant. It will prove convenient to introduce the variables

(19) $$x\equiv T_{h}-T_{h,p}\;\mathrm{and}\;y\equiv T_{c,p}-T_{c}$$

which is identical to the notation introduced by the original treatment by Curzon and Ahlborn [10].

After a few lines of simple algebra, we obtain

(20) $$P\left(x,y\right)=\frac{\alpha \beta \;xy\;\left[\left(T_{h}-x\right)+\left(T_{c}+y\right)\right]}{\gamma \left[\alpha \;x\;\left(T_{h}-x\right)+\beta \;y\;\left(T_{c}+y\right)\right]}\;,$$

where we once again employed Equations (4) and (7). The maximum of P(x,y) is determine using standard calculus, namely solving $\partial_{x}P\left(x,y\right)=0$ and $\partial_{y}P\left(x,y\right)=0$ for *x* and *y*.

Denoting the solutions by $x^{⁎}$ and $y^{⁎}$, we find

(21) $$x^{⁎}=\frac{\sqrt{\beta }}{\sqrt{\alpha }+\sqrt{\beta }}\;\left(T_{h}-\sqrt{T_{h}T_{c}}\right)\;\mathrm{and}\;y^{⁎}=\frac{\sqrt{\alpha }}{\sqrt{\alpha }+\sqrt{\beta }}\;\left(-T_{c}+\sqrt{T_{h}T_{c}}\right)\;.$$

Substituting these solutions into the expression for the efficiency (17) and simplifying the expression we finally obtain

(22) $$\eta =1-\sqrt{\frac{T_{c}}{T_{h}}}\;.$$

That is, the efficiency at maximal power of an endoreversible Stirling engine operating with a one-component plasma as working medium is given by the Curzon–Ahlborn efficiency. Our result (22) corroborates earlier findings for classical ideal gases [32,33,34]. Thus, engines operating in endoreversible Stirling cycles with one-component plasmas and classical ideal gases have the same efficiency.

### 2.3. Generalization to Second Order Virial Expansion

Before we move on to more intricate working mediums, we emphasize that the present analysis is entirely based on the fact that the caloric equation of state is of the form of an ideal gas (3). In particular, we did not need to require the mechanical equation of state to be given by the ideal gas law, nor did we need the full fundamental relation.

Interestingly, equations of state of the form (3) are *not* restricted to *classical* ideal gases. For instance, classical harmonic oscillators [16] and rubber bands [37] are also described by equations of state that are linear in temperature (and independent of volume).

For the present purposes, it is even more interesting to observe that the van der Waals gas is described by [37]

(23) $$E=a_{0}\;T-\frac{b_{0}}{V^{2}}\;,$$

where $a_{0}$ and $b_{0}$ are phenomenological constants. It is a simple exercise to show that the van der Waals equation of state is obtained from the virial expansion in second order [38]. Since the dependence on the volume *V* in Equation (23) is only additive, the changes in entropy during isochoric strokes remain identical to above (14). Consequently, we obtain the same expressions for efficiency (17), power (20), and efficiency at maximal power (22).

It is worth emphasizing that for many real plasmas virial coefficients have been determined; see, for instance, [39,40,41,42,43]. Our present results remain valid for any endoreversible Stirling cycle for plasma, for which a description of up to second order in the virial expansion is a good description.

## 3. Relativistic Electron–Positron–Photon Plasma

In the preceding section we analyzed the performance of endoreversible plasma engines that are described by equations of state which are (i) linear in temperature and (ii) at most additive in volume. The situation becomes more involved if these conditions are not met.

To this end, we conclude the analysis with another type of plasma, namely the relativistic electron–positron–photon plasma. Rather recently, it was shown in Ref. [36] that in the high-temperature limit, the corresponding caloric equation of state can be written as the familiar photonic gas law [37]:

(24) $$E=\epsilon VT^{4}\;,$$

where ϵ is a constant collecting natural constants, such as the speed of light, the electron mass, and *ℏ*; see Ref. [36]. In principle, we could also work with the more involved expression at finite temperature, but this would necessitate a fully numerical treatment. We choose to continue with Equation (24) as it allows an almost entirely analytical treatment.

Note that Equation (24) does not have the simple, linear form of Equation (3) that we exploited above. Therefore, the natural question arises whether engines with photonic plasmas operate at higher or lower efficiency than those with one-component plasmas. Note that for the same question, but for Otto cycles it was found that photonic gases significantly outperform classical ideal gases [25].

### 3.1. Endoreversible Stirling Cycle

**Figure 2:**
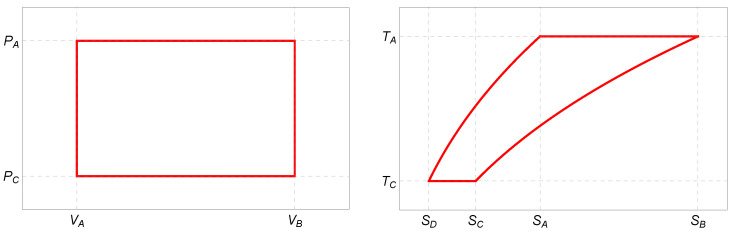
Schematic PV- and TS-diagrams of the Stirling cycle for the photonic gas (24) as a working medium.

To answer this question, we repeat the construction of the endoreversible Stirling cycle paying special attention to the required modifications. In Figure 2, we plot the corresponding PV- and TS-diagrams schematically.

#### 3.1.1. A→B: Isothermal Expansion

As before, we assume that during the hot isotherm the plasma is slightly colder than the hot heat reservoir, and that the heat flux is given by Fourier’s law. Hence, we can write again $Q_{AB}=\alpha t_{AB}x$. However, in this case we can no longer assume that the internal energy is constant, as Equation (24) depends multiplicatively on the volume. Hence, we have to write for the work

(25) $$W_{AB}=\Delta E_{AB}-Q_{AB}=\epsilon \;T_{h,p}^{4}\;\left(V_{B}-V_{A}\right)-\alpha t_{AB}x\;,$$

which explicitly depends on the change in volume as well as the stroke time.

It is a standard exercise [37] to show that the entropy of the photonic gas reads

(26) $$S=\frac{4}{3}\epsilon VT^{3}\;.$$

And, as discussed above, for isothermal processes, we simply have $Q_{AB}=T_{h,p}\;\Delta S_{AB}$. Therefore, we can also write

(27) $$\alpha t_{AB}x=\frac{4}{3}\;T_{h,p}^{4}\;\left(V_{B}-V_{A}\right)\;,$$

which will become useful shortly.

#### 3.1.2. B→C: Isochoric Cooling

For the isochoric strokes, we again have that $W_{BC}=0$ and

(28) $$Q_{BC}=\Delta E_{BC}=\epsilon V_{B}\left(T_{c,p}^{4}-T_{h,p}^{4}\right)\;.$$

However, as seen above, the work and heat of the isochoric strokes will not be required for further analysis.

#### 3.1.3. C→D: Isothermal Compression

In complete analogy to the hot isotherm, we now can write $Q_{CD}=-\beta t_{CD}y$, and

(29) $$W_{CD}=\Delta E_{CD}-Q_{CD}=-\epsilon \;T_{c,p}^{4}\;\left(V_{B}-V_{A}\right)-Q_{CD}\;.$$

Moreover, again employing the expression for the entropy (26) and $Q_{CD}=T_{c,p}\;\Delta S_{CD}$ we also have

(30) $$\beta t_{CD}y=\frac{4}{3}\;T_{c,p}^{4}\;\left(V_{B}-V_{A}\right)\;.$$

#### 3.1.4. D→A: Isochoric Heating

For completeness, we also collect the work, $W_{DA}=0$, and heat,

(31) $$Q_{DA}=\Delta E_{DA}=\epsilon V_{A}\left(T_{h,p}^{4}-T_{c,p}^{4}\right)\;,$$

during the isochoric heating stroke.

#### 3.1.5. Endoreversible Efficiency

Before we start analyzing the power, we again first derive an expression for the efficiency:

(32) $$\eta \equiv -\frac{W_{\mathrm{cyc}}}{Q_{AB}}=-\frac{W_{AB}+W_{CD}}{Q_{AB}}\;.$$

Substituting Equations (25), (27), (29), and (30) into the definition (32) we obtain,

(33) $$\eta =\frac{1}{4}\left[1-{\left(\frac{T_{c,p}}{T_{h,p}}\right)}^{4}\right]\;.$$

**Figure 3:**
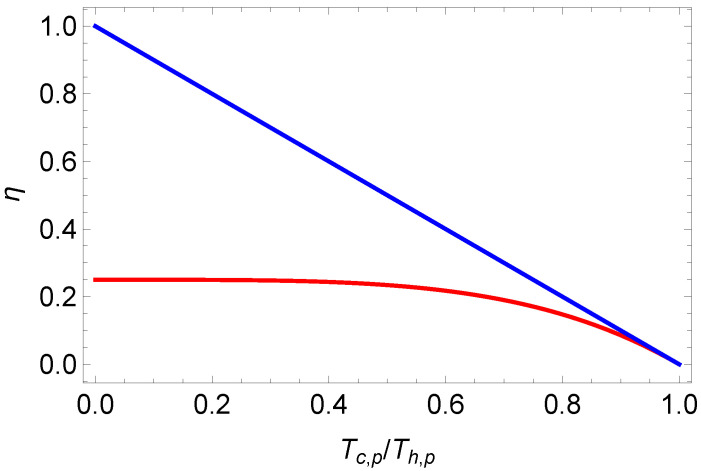
Endoreversible Stirling efficiency for the one-component plasma (22) (blue line) and the photonic gas (33) (red line).

Comparing the latter result (33) with the endoreversible efficiency for one-component plasmas (17) we note that endoreversible Stirling efficiency for photonic gases is always smaller than Equation (17); cf. Figure 3.

### 3.2. Efficiency at Maximal Power

The obvious question now is what this means for the efficiency at maximal power. To this end, we again consider the power output

(34) $$P=\frac{Q_{AB}+Q_{CD}}{\gamma \;(t_{AB}+t_{CD})}\;,$$

which in the present case can be written as

(35) $$P\left(T_{c,p},T_{h,p}\right)=\frac{\alpha \beta }{4\gamma }\frac{\left(T_{h}-T_{h,p}\right)\left(T_{c}-T_{c,p}\right)\left(T_{h,p}^{4}-T_{c,p}^{4}\right)}{\beta T_{h,p}^{4}\left(T_{c}-T_{c,p}\right)-\alpha T_{c,p}^{4}\left(T_{h}-T_{h,p}\right)}\;.$$

We immediately observe that Equation (35) is significantly more involved than the power for one-component plasmas (20), and thus we have to resort to a numerical analysis.

**Figure 4:**
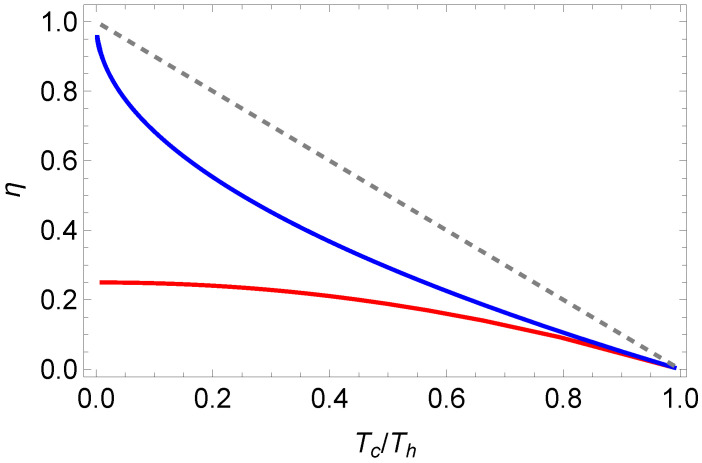
Efficiency at maximal power for the photonic equation of state (24) (red line) together with the Curzon–Ahlborn efficiency (22) (blue line), and the Carnot efficiency, $\eta_{C}=1-T_{c}/T_{h}$ (gray, dashed line). Parameters are α=1, β=1, and γ=1.

In Figure 4, we depict the solution for one set of parameters. We also observe that at maximal power, the efficiency for photonic gases is significantly below the Curzon–Ahlborn efficiency (22). We emphasize again that this is in contrast to endoreversible Otto cycle, in which the photonic case has a higher efficiency [25].

## 4. Concluding Remarks

In the present work, we analyzed the efficiency at maximal power of endoreversible Stirling cycles. As working mediums, we considered one-component plasmas with caloric equations of state, extensions in second order virial expansion, and photonic gases. We found that for all thermodynamics systems, whose caloric equation of state only depends linearly on temperature and at most additively on volume, the efficiency at maximal power is given by the seminal Curzon–Ahlborn efficiency. Interestingly, the efficiency for photonic working mediums is significantly smaller, which is in stark contrast to endoreversible Otto cycles.

Particular emphasis was put on a comprehensive, self-contained, and pedagogical presentation of the treatment. While our work is purely theoretical and it is unlikely that our results will lead directly to the experimental realization of a practically useful plasma Stirling engine, the conceptual mathematical steps of our analysis could be applied to more realistic scenarios. The only necessary “ingredient” is the caloric equation of state for any considered plasma, which then step-for-step leads to the corresponding expression for the efficiency at maximal power.

## Data Availability

The original contributions presented in this study are included in the article. Further inquiries can be directed to the corresponding authors.

## Appendix A. Equal Strokes Times—Trivial Case

In this appendix, we briefly discuss a trivial case for the one-component plasma (3). As previously conducted for Otto [16] and Brayton [17] cycles, one is tempted to make a “simplifying” assumption. Namely, to set the stroke times equal to each other, $\tau \equiv t_{AB}=t_{CD}$. In this case, we can set Equation (18) as

(A1) $$P=\frac{1}{2\gamma }\;\left(\alpha \;x-\beta \;y\right)\;,$$

which is linear in *x* and *y*. In this case, we can determine the maximal power simply from the maximal and minimal values of *x* and *y*, respectively. Recognizing that at no instant the plasma can be colder than the cold reservoir, we can write

(A2) $$x_{\max}=T_{h}-T_{c}\;\mathrm{and}\;y_{\min}=0\;.$$

Substituting the latter into Equation (11) we obtain

(A3) $$\eta =1-\frac{\beta }{\alpha }\frac{y_{\min}}{x_{\max}}=1\;.$$
